# Discharge destinations for young people with a first episode of psychosis after attending an early intervention for psychosis service

**DOI:** 10.1177/00048674231172404

**Published:** 2023-05-10

**Authors:** Brian O’Donoghue, Andrew Thompson, Patrick McGorry, Ellie Brown

**Affiliations:** 1 Department of Psychiatry, University College Dublin, Dublin, Ireland; 2 Orygen, Parkville, VIC, Australia; 3 Centre for Youth Mental Health, The University of Melbourne, Parkville, VIC, Australia; 4 Elm Mount Unit, St Vincent’s University Hospital, Dublin, Ireland

**Keywords:** Psychosis, schizophrenia, early intervention, discharge

## Abstract

**Objective:**

Early intervention for psychosis services result in superior outcomes in the domains of symptomatic and functional recovery, hospitalisation and employment compared to standard services; however, the optimal duration of care with these services is unknown. Knowledge on the discharge destinations, specifically the proportion discharged to high- and low-intensity services, could provide insights into the proportion of who may require a longer tenure of care. This study aimed to determine (1) the discharge destinations from early intervention for psychosis services and (2) baseline and intra-episode factors associated with discharge to the secondary care/adult mental health service.

**Methodology:**

This study was conducted at the Early Psychosis Prevention and Intervention Centre in Melbourne and included all young people treated by the service with a first episode of psychosis over a 6-year period. Discharge destinations were categorised according to high-intensity services, namely, secondary mental health care, or lower intensity services, such as private practitioners or primary care.

**Results:**

A total of 1101 young people with a first episode of psychosis were included in the study, of whom 58.8% were male and the median age was 20.0 years (interquartile range: 17–22). After a median of 95.4 weeks (interquartile range: 66.7–105.7), 36.6% were discharged to the adult mental health services, which was associated with being not in employment, education or training at presentation (odds ratio = 1.71, 95% confidence interval [1.23, 2.37]); experiencing a relapse (odds ratio = 1.76, 95% confidence interval [1.24, 2.49]); and being admitted to a mental health unit (odds ratio = 3.98, 95% confidence interval [2.61, 6.09]). Young people who lived with their parents were less likely to be discharged to secondary care services (odds ratio = 0.52, 95% confidence interval [0.37, 0.73]), as were those who were achieving symptomatic remission within 12 weeks (odds ratio = 0.60, 95% confidence interval [0.43, 0.83]). Migrant status and the duration of untreated psychosis were not associated with discharge destination.

**Conclusion:**

These findings indicate that there is a sizable, identifiable minority who may benefit from a longer episode of care with early intervention for psychosis services.

## Introduction

Early intervention for psychosis services results in superior outcomes in the domains of symptomatic and functional recovery, hospitalisation and returning to employment (Correll et al., 2018). This is achieved by providing a broad suite of interventions consisting of pharmacotherapy, psychosocial treatments, family support, vocational training and physical health services (Stavely et al., 2013). The aim of these intensive, community-based and hospital supports is to assist an individual to recover from, and maintain remission from, their first episode of psychosis (FEP) and most importantly, to return to their premorbid level of functioning (Simonsen et al., 2017). Early Intervention for Psychosis services tend to have a defined period of care, usually for at least 2 years, as this was initially deemed to be the critical period (Crumlish et al., 2009), and also the interventions are very intensive.

The optimal time that early intervention services should provide care has been investigated in three randomised trials. In Hong Kong, it was found that an additional year in early intervention services resulted in superior outcomes, in relation to functioning, negative and depressive symptoms, compared to step down care (Chang et al., 2015). While in Denmark, extending early intervention services by an additional 3 years to a total of 5 years was associated with some additional benefits, such as improved engagement, working alliance and higher satisfaction with services, although there were no benefits in negative symptoms, which was the primary outcome (Albert et al., 2017). In Canada, extending early intervention services to 5 years resulted in longer remission of both positive and negative psychotic symptoms and superior engagement (Malla et al., 2017). Considering these findings, it is possible that there may be a sub-group of individuals with an FEP who require a longer period of care with early intervention services and identifying these individuals from the outset could facilitate earlier targeted treatment. One method to identify this group who may require a longer period of care is to examine the discharge destinations after the tenure of care with the early intervention for psychosis service as a real-world marker of medium-term outcome.

The potential discharge destinations available after the episode of care within an early intervention for psychosis service is likely to vary in different countries or even different jurisdictions. However, discharge destinations could be broadly categorised into the highest intensity service available, which is likely to be the secondary care services/adult mental health services/specialist youth mental health services, or a lower intensity service, such as primary care (including headspace in Australia) or private practitioners. In two separate studies in the United Kingdom, it was found that approximately half of individuals with an FEP were discharged to primary care (Ahmed et al., 2019; Phillipson et al., 2014). However, the largest study to date, also from the United Kingdom, found that the vast majority of individuals, 83.5%, were discharged to primary care after their episode of care with the early intervention service (Puntis et al., 2018). Individuals who were admitted to hospital during their episode of care were nearly twice as likely to be discharged to the adult mental health services post early intervention (Puntis et al., 2018). This research has been confined to one country and also a limited number of factors associated with the discharge destination were examined in each individual study.

To address this gap in knowledge, this study aimed to determine (1) the discharge destinations for young people after their tenure of care with an early intervention for psychosis service and (2) the factors associated with discharge to the secondary care/adult mental health service, specifically factors present at baseline and during the episode of care.

## Methods

### Setting

This study was undertaken at the Early Psychosis Prevention and Intervention Centre (EPPIC) service, which is a specialist programme within Orygen, a youth mental health service based in the North-Western area of Melbourne, Australia (McGorry et al., 1996). EPPIC provides care for all young people aged between 15 and 24 years diagnosed with an FEP. The treated incidence rate of FEP in the catchment area has been found to be 123 per 100,000 at risk population (Eaton et al., 2019), and there are no other public mental health services in the catchment area that provide treatment to young people with an FEP. The EPPIC service provides comprehensive case-management and in addition to psychiatric care, offers psychological therapy, namely, cognitive-behavioural therapy for psychosis; family psychoeducation and family-peer support; and vocational interventions, such as individual placement support, physical health interventions and group-based interventions. Further family-based interventions may be provided if indicated, such as family therapy. Young people attending the EPPIC service receive up to 2 years of treatment with the early intervention for psychosis service, unless they are aged less than 16 at the time of presentation, as in these cases, they can attend the service up until the age of 18. While there are private psychiatrists located within the catchment area, it is unlikely that they would offer care to people with an FEP, as the EPPIC service is well established, and these individuals are typically directed towards the public service; however, it is possible that cases were missed as they may have sought treatment privately.

### Services available post discharge

Within the catchment area of the current cohort, the public secondary mental health service, also termed the adult mental health service, was the most intensive service available. Individuals referred to this service typically received care from a consultant psychiatrist and case-manager within a multidisciplinary team. In contrast to early intervention services, these services do not typically have a limit on the length of time an individual remains in care. There were also a number of less intensive services available, and these included a private psychiatrist or private psychologist or a primary care service, such as a general practitioner or a *headspace* service, a designated youth mental health primary health service.

### Participants

This study included all individuals who received treatment at the EPPIC service between 1 January 2011 and 31 December 2016. An FEP was operationalised as having at least one positive psychotic symptom daily, for at least 1 hour, for at least 1 week. There were no exclusion criteria for the EPPIC service, and individuals with a concurrent diagnosis of substance abuse or personality disorders were included within the service.

### Design and procedure

This was a naturalistic cohort study whereby data were recorded prospectively but collected retrospectively from clinical files. Researchers extracted data from clients’ paper and electronic medical records using a specifically designed audit tool. Clinical records include data compiled during their care at Orygen from initial assessment reports, outpatient notes, inpatient notes (if applicable), clinical review meetings and discharge letters.

### Measures and instruments

Diagnoses were determined by the treating consultant psychiatrist and were categorised as either non-affective (schizophrenia, schizophreniform disorder, substance-induced psychotic disorder, delusional disorder, brief psychotic disorder and psychotic disorder not otherwise specified [NOS]) or affective (schizoaffective disorder, bipolar disorder with psychotic features, major depressive disorder with psychotic features). The duration of untreated psychosis (DUP) was determined by the clinical assessment at intake into the service. All admissions, including to other psychiatric inpatient units, were systematically recorded in the clinical files. The severity of psychotic symptoms was assessed and rated at baseline, and at three monthly intervals thereafter, and positive symptoms were rated using the short form Scale for the Assessment of Positive Symptoms (SAPS) (Alonso et al., 2008), which specifically measured hallucinations, delusions, bizarre behaviour and thought disorder. Remission was defined as a score of 2 or less on all these items in the short form SAPS, and relapse was defined as the return of any positive symptom (defined as a score of 3 or more on any item). Inter-rater reliability was assessed across five different participants, and on all positive psychotic symptom items, inter-rater agreement ranged from 80% to 100%. The achievement of remission by 12 weeks was selected as a predictor variable, as this timepoint has been demonstrated to a long-term predictor of positive outcomes in FEP (Dazzan et al., 2020).

### Statistical analysis

Binary logistic regression was conducted to identify predictor variables associated with the binary outcome of either discharge to the adult mental health services (high intensity) or a lower intensity health service. Initially, the association between the individual predictor variable and the outcome variable was conducted, and then any variables that were associated with the outcome at a significance level of *p* < 0.10 were entered into a multivariate logistic regression model. Three regression models were performed. Model I examined the association between demographic and clinical factors that were present at the time of initial presentation. Model II examined the association of clinical factors during the episode of care with the outcome of discharge destination, and Model III examined all of these factors together.

### Ethical approval

This study was approved by the Royal Melbourne Human Research and Ethics Committee (reference: QA2018034). In order for a fully representative cohort to be included in the study, a waiver of individual consent was granted for individuals attending the EPPIC service during the study period, including those aged 15–17 years.

## Results

### Description of participants

A total of 1220 young people presented with an FEP during the 6-year study period and of these, 1.4% (*n* = 17) had not been discharged by the end of the period of data collection, and there was missing data for a further 8.4% (*n* = 102) in relation to the discharge destination. Therefore, there was complete data for 90.2% (*n* = 1101) of the total cohort, and of these, 58.8% (*n* = 647) were male, the median age at presentation was 20.0 years (interquartile range [IQR]: 17–22), 25.4% (*n* = 280) were first generation migrants and 43.1% (*n* = 475) had a diagnosis of a schizophrenia spectrum disorder. The demographic and clinical characteristics of the cohort are presented in Table 1.

**Table 1.** Demographic and clinical characteristics of the cohort at the time of presentation according to discharge destination.

	Total sample (*n* = 1101)	Adult mental health service (*n* = 403)	Lower intensity service (*n* = 698)	OR (95% confidence intervals)	*p*
	Median (IQR)	Median (IQR)	Median (IQR)		
Age	20.0 (17–22)	20.0 (18–22)	19.0 (17–22)	1.03 (0.99–1.08)	0.159
	% (*n*)	% (*n*)	% (*n*)		
Sex – % male	58.8 (647)	64.0 (258)	55.7 (389)	1.41 (1.10–1.82)	0.007
Marital status – % never married	95.9 (1035)	95.7 (377)	96.1 (658)	0.91 (0.49–1.69)	0.910
Living arrangements – % with parents	67.8 (746)	60.3 (243)	72.1 (503)	0.59 (0.45–0.76)	<0.001
Not in employment, education or training	46.1 (503)	58.4 (233)	39.0 (270)	2.19 (1.71–2.82)	<0.001
Family history of psychosis					
First-degree relative	18.2 (200)	21.3 (86)	16.3 (114)	1.39 (1.02–1.90)	0.038
Second-degree relative	25.4 (280)	27.5 (111)	24.2 (169)	1.38 (0.98–1.93)	0.065
Cultural background					
Migrant	25.4 (280)	27.5 (111)	24.2 (169)	1.19 (0.90–1.57)	0.222
Aboriginal or Torres Strait Islander	2.8 (31)	4.0 (16)	2.2 (15)	1.88 (0.92–3.84)	0.084
Diagnosis at baseline					
Schizophrenia	17.8 (196)	26.8 (108)	12.6 (88)	-	–
Schizophreniform disorder	19.8 (218)	17.1 (69)	21.3 (149)		
Schizoaffective disorder	5.5 (61)	8.9 (36)	3.6 (25)		
Delusional disorder	1.4 (15)	1.0 (4)	1.6 (11)		
Drug-induced psychosis	11.3 (124)	9.2 (37)	12.5 (87)		
Bipolar affective disorder	13.0 (143)	11.2 (45)	14.0 (98)		
Depression with psychosis	8.9 (98)	7.2 (29)	9.9 (69)		
Psychotic disorder NOS	14.8 (163)	11.9 (48)	16.5 (115)		
Brief psychotic disorder	2.4 (26)	1.5 (6)	2.9 (20)		
Not differentiated	5.2 (57)	5.2 (21)	5.2 (36)		
Diagnostic groupings					
Non-affective disorder	71.1 (742)	71.2 (272)	71.0 (470)	1.01 (0.77–1.33)	0.943
Affective disorder	28.9 (302)	29.0 (192)	28.8 (110)		
Substance misuse					
Any co-morbid substance use	38.5 (413)	44.3 (175)	35.2 (238)	1.47 (1.14–1.89)	0.003
Alcohol	17.2 (189)	17.9 (72)	16.8 (117)	1.08 (0.78–1.49)	0.640
Cannabis	51.6 (568)	58.6 (236)	47.6 (332)	1.56 (1.22–2.00)	<0.001
Amphetamine	27.2 (299)	35.2 (142)	22.5 (157)	1.88 (1.43–2.46)	<0.001
Clinical factors					
Admission at time of presentation	50.3 (548)	57.0 (227)	46.4 (321)	1.53 (1.20–1.97)	<0.001
Involuntary admission at time of presentation	34.3 (354)	40.7 (151)	30.8 (203)	1.54 (1.19–2.01)	<0.001
	Median (IQR)	Median (IQR)	Median (IQR)		
Duration of untreated psychosis (weeks)	8.0 (2–40)	8.0 (2–52)	8.0 (2–36)	1.00 (0.99–1.00)	0.761

OR: odds ratio; IQR: interquartile range; NOS: not otherwise specified.

### Rates and characteristics by discharge destination

After a median of 95.4 weeks (IQR: 66.7–105.7), 36.6% (*n* = 403) of the young people were discharged to the adult mental health services, while 63.4% (*n* = 698) were discharged to a lower intensity service, which included primary care (46.3%), private psychiatrist (11.7%) and private psychologist (5.4%).

### Predictors of discharge destinations

#### Baseline characteristics

On univariate analysis, the following factors that were present at the time of presentation were associated with subsequent discharge to the adult mental health service: male sex (OR = 1.41, 95% CI [1.10, 1.82]); being not in employment, education or training (NEET) (OR = 2.19, 95% CI [1.71, 2.82]); having a first degree relative with a psychotic disorder (OR = 1.39, 95% CI: [1.71, 2.82]); cannabis use (OR = 1.56, 95% CI [1.22, 2.00]); metamphetamine use (OR = 1.88, 95% CI [1.43, 2.46]); and being admitted to hospital (OR = 1.53, 95% CI [1.20, 1.97]). Young people who were living with their parents were less likely to be discharged to adult mental health services (OR = 0.59, 95% CI [0.45, 0.76]). When all of these factors were combined in a multivariate regression model (Table 3), the following factors remained significant: living with parents (OR = 0.61, 95% CI [0.47, 0.80]), being NEET (OR = 1.79, 95% CI [1.38, 2.34]), having a first degree relative with a psychotic disorder (OR = 1.41, 95% CI [1.02, 1.95]), methamphetamine use (OR = 1.54, 95% CI [1.12, 2.11]) and admission to hospital (OR = 1.45, 95% CI [1.12, 1.88]).

#### Clinical factors during episodes of care

On univariate analysis, the following factors were associated with discharge to the adult mental health services: experiencing a relapse during the episode of care (OR = 2.77, 95% CI [2.15, 3.57]; being admitted to hospital during the episode of care, excluding at presentation, (OR = 5.16, 95% CI [3.94, 6.75]) and being admitted involuntarily (also excluding at presentation) (OR = 3.93, 95% CI [2.97, 5.20] (Table 2). Achieving remission within 12 weeks of presentation was associated with a reduced risk of being discharged to the adult mental health service (OR = 0.54, 95% CI [0.41, 0.72]); When all of these factors were examined together in a multivariate regression model, all of the factors remained significantly associated with the discharge destination, except being involuntarily admitted during the episode of care (Table 3).

**Table 2.** Clinical factors during episodes of care associated with discharge destination.

	Total sample (*n* = 1101)	Adult mental health service (*n* = 403)	Lower intensity service (*n* = 698)	OR (95% CI)	*p*
Remission achieved within 12 weeks	65.9 (594)	57.0 (184)	70.9 (410)	0.54 [0.41, 0.72]	<0.001
Relapse occurred	39.0 (429)	54.3 (219)	30.1 (210)	2.77 [2.15, 3.57]	<0.001
Admitted, excluding at presentation	*46.7 (510)*	71.4 (284)	32.6 (226)	5.16 [3.94, 6.75]	<0.001
Involuntarily admitted, excluding at presentation	26.8 (295)	44.2 (178)	16.8 (117)	3.93 [2.97, 5.20]	<0.001

OR: odds ratio; CI: confidence interval.

**Table 3.** Multivariate analysis of baseline and intra-episode of care factors associated with discharge to secondary care services.

	Models I and II			Model III		
	OR	95% CI	*p*	OR	95% CI	*p*
Demographic and clinical characteristics at baseline						
Sex – male	1.31	[0.99, 1.72]	0.056	1.37	[0.99, 1.90]	0.061
Living with parents	0.61	[0.47, 0.80]	<0.001	0.52	[0.37, 0.73]	<0.001
Not in employment, education or training	1.79	[1.38, 2.34]	<0.001	1.71	[1.23, 2.37]	0.002
First-degree relative with psychotic disorder	1.41	[1.02, 1.95]	0.040	1.49	[0.99, 2.21]	0.052
Cannabis abuse	1.04	[0.77, 1.41]	0.784	1.02	[0.72, 1.46]	0.91
Methamphetamine abuse	1.54	[1.12, 2.11]	0.007	1.30	[0.88, 1.91]	0.183
Admission to hospital	1.45	[1.12, 1.88]	0.005	1.19	[0.87, 1.64]	0.283
Clinical factors during episode of care						
Remission achieved within 12 weeks	0.63	[0.46, 0.86]	0.003	0.60	[0.43, 0.83]	0.002
Relapse occurred	1.72	[1.24, 2.39]	0.001	1.76	[1.24, 2.49]	0.002
Admitted, excluding at presentation	3.40	[2.29, 5.07]	0.001	3.98	[2.61, 6.09]	<0.001
Involuntarily admitted, excluding at presentation	1.43	[0.97, 2.12]	0.075	1.13	[0.74, 1.72]	0.571

OR: odds ratio; CI: confidence interval.

Model I examines the association between demographic and clinical factors that were present at the time of initial presentation with the outcome of discharge destination. Model II examines the association of clinical factors during the episode of care with the outcome of discharge destination. Models I and II used univariate analysis and Model III examined all of these factors together with multivariate analysis.

#### Multivariate analysis

Baseline and intra-episode factors that were associated with the discharge destination on univariate analysis were examined together in a multivariate analysis (Table 2 – Model III), and when examined together, five factors remained statistically significant, two were associated with a reduced odds of being discharged to the adult mental health service, namely: living with parents (OR = 0.52, 95% CI [0.37, 0.73]) and achieving remission within 12 weeks of presentation (OR = 0.60, 95% CI [0.43, 0.83]). Three factors were associated with an increased odds of being discharged to adult mental health services, namely being NEET at presentation (OR = 1.71, 95% CI [1.23, 2.37]), experiencing a relapse (OR = 1.76, 95% CI [1.24, 2.49]) and being admitted to hospital during the episode of care (OR = 3.98, 95% CI [2.61, 6.09]).

## Discussion

### Summary of findings

In this naturalistic follow-up study of a large cohort of young people with an FEP, it was found that after an episode of care with an early intervention for psychosis service of a median of nearly 2 years, 36.6% of young people were discharged to a secondary care service/adult mental health service, and this was associated with being NEET at the time of presentation, experiencing a relapse and having an inpatient admission during the episode of care. Achieving remission within 12 weeks of presentation and living with parents were associated with a reduced odds of being discharged to a secondary care service.

These findings are in contrast to the studies in the United Kingdom, two of which found that nearly half of individuals were discharged to secondary care (7, 8), while the other found that less than one in five were discharged to secondary care (9). This is understandable, considering that it is likely that alternative discharge destinations, such as the availability and relative affordability of private practitioners, will vary in different jurisdictions. However, one of the common themes across studies is that an admission during the care with the early intervention service is strongly associated with discharge to the secondary care services. Admission to hospital could be conceived as being a proxy indicator for more severe illness, or episodes that had more acuity or risks involved or limited social or family supports. However, it may also be that step down services, such as private practitioners, are reluctant to accept referrals for individuals who have had admissions during their original episode of care, due to a concern that they may have to arrange a subsequent admission. Our study design could not determine this, but further quantitative work into potential barriers to individuals being referred to primary care or other lower intensity services are needed.

### Strengths and limitations

While there are several strengths to this study, such as the large sample size and the ability to control for multiple variables, the findings need to be considered within the limitations of the study. One of the main limitations is that information was only available for the time during the tenure of care, usually 2 years. Therefore, we did not have data available pertaining to the situation in which a young person who was initially discharged to a lower intensity service was subsequently referred to secondary care, such as in the event of a relapse. There were also a few factors which were reliant on self-report, such as substance misuse, which may have been under-estimated by this method. A further limitation of this study is that we could only examine the demographic and clinical characteristics that were collected at the time of the study, and there may have been other factors, such as cognitive deficits or poor functioning, that contributed to the decision to discharge the individual to the secondary care services. In addition, while we identified factors associated with discharge to secondary care services, we do not know the exact reason. For example, being NEET could be an indicator of poor functioning, or it may be that individuals do not have resources to pay for a private practitioner. These data were also relating to individuals presenting with an FEP between 2011 and 2016, and there were missing data for nearly 10% of the cohort, which may affect the generalisability of results.

### Clinical implications

There are a number of important clinical implications from the study. First, as discharge to the secondary care services could be considered a proxy measure for the need for an equivalent level of services, this provides us with an estimate of the proportion of the individuals with an FEP who may require longer than a 2-year tenure of care with the early intervention for psychosis service. The aforementioned randomized controlled trials that examined the effectiveness of extending the tenure of care with the early intervention service included all individuals who had initially presented with an FEP (Albert et al., 2017; Chang et al., 2015; Malla et al., 2017). Another approach that could be considered would be to evaluate the effectiveness of extending the tenure of care in individuals who have the factors associated with a poorer outcome, such as relapse, admission and being NEET. Although, it needs to be acknowledged that another benefit of extending early intervention for psychosis is to maintain the recovery that was achieved in the initial episode of care with the service. Therefore, while there may be an advantage in identifying individuals who may require longer treatment with the early intervention service, this should not be to the detriment of those with positive prognostic factors. Their period of care should not be shortened, as a long period of sustained recovery is crucial prior to an individual being discharged to a lower intensity service. However, individuals who have achieved remission, with positive prognostic factors, could remain with the early intervention service for a period of active monitoring and would not necessarily have to avail of the intensive interventions. The advantage of this approach is that if they experienced any re-emergence of symptoms, they could quickly access the appropriate service and access more intensive components of the service should the need arise. This needs to be based on valid markers of prognostic outcome and needs to be flexible to allow rapid step-up care if required.

The rationale for youth-based mental health services is that they cover the peak age at onset for mental health disorders and therefore reduce the need for individuals to transition between Child & Adolescent mental health services and adult services. Therefore, reducing transitions between services is a core objective of youth mental health services. This study has demonstrated that 36% of young people had to transition to the adult mental health services after their tenure of care with the early intervention service. Another approach would be for individuals to be able to remain with the youth mental health service up to the age of 24 or 25 years, regardless of the age at the time of entry to the service. This would facilitate young people continuing to receive care within the environment of a youth mental health and reduce transitions to other services. It is planned to evaluate such a service model within EPPIC. Another way to explore these issues further is through the establishment of Clinical Trial Registries of individuals experiencing an FEP with subsequent data linkage, which allows for longer term follow-up, and subsequent enhanced care planning. This is currently underway within the EPPIC programme through the Australian Early Psychosis Collaborative Consortium (AEPCC).

An encouraging aspect of the findings of this study is that some of the factors that were associated with discharge to secondary care are potentially modifiable. Two of the core objectives of early intervention services are to achieve timely remission of psychotic symptoms and prevent relapse (Stavely et al., 2013). Within a period of 12–16 weeks, it is possible to have trials of two different antipsychotic medications, and therefore even at this stage, treatment resistance could be identified (Lally et al., 2016) and work has commenced on a prediction tool to identify individuals with treatment resistance (Farooq et al., 2022). At this point, individuals with a diagnosis of treatment-resistant schizophrenia could be considered for clozapine. In addition, the use of long-acting injectable antipsychotic medications in FEP have been shown to improve adherence, remission of symptoms and prevention of relapse (Lian et al., 2022). Although, these interventions that could be delivered to achieve early remission of symptoms could also lead to an ongoing need for secondary mental health services, as they may be the only services that will prescribe and monitor clozapine or long-acting injectable antipsychotic medications.

In addition, early intervention for psychosis services have been shown to have an overall effect in reducing relapse in FEP (Alvarez-Jiménez et al., 2011), while the further addition of family-based interventions and a relapse prevention intervention can further reduce the risk of relapse (Camacho-Gomez and Castellvi, 2020; Gleeson et al., 2013). Therefore, there could be considerable gains by ensuring that these interventions are provided early during the disorder. In this study, the DUP was not related to the discharge destination; however, the median DUP was 8 weeks, as the EPPIC early intervention service continues to provide educational campaigns to maintain a low DUP (Krstev et al., 2004). Therefore, it is possible that this limited variance in the DUP resulted in it not having an impact in this cohort, but it would still warrant examination in other cohorts with newly established early intervention services.

## Conclusion

Just over one-third of young people attending an early intervention for psychosis service were discharged to the secondary care services, and these individuals were more likely to be unemployed or not in education or training at the time of presentation, experience a relapse and be admitted to hospital. This indicates that there is likely to be a potentially identifiable sub-group of individuals with an FEP who require a longer tenure of care with early intervention services.
